# Digital Health Support for Cataract Surgery With the Sharp Health Companion CareKit App: Randomized Controlled Trial

**DOI:** 10.2196/78710

**Published:** 2026-04-29

**Authors:** Tommy S Korn, Armand Assissini, Lesli Ann Slater, Janet Holland, Lauren Doone, Elvis Lam, Joy Fox, Kristina L Greenwood, Eric Linebarger

**Affiliations:** 1 Department of Ophthalmology Sharp HealthCare Sharp Rees-Stealy Medical Group San Diego, CA United States; 2 Sharp HealthCare San Diego, CA United States

**Keywords:** CareKit, cataract surgery, digital health, medication adherence, mHealth, mobile health, older adults, patient education, perioperative care, surgical outcomes, telemedicine

## Abstract

**Background:**

Cataract surgery is the most frequently performed surgery worldwide, crucial for restoring sight in millions. The COVID-19 pandemic and an aging population have increased barriers to timely surgery. Missed preoperative instructions and poor adherence to postoperative care contribute to surgery cancellations, delays, and potential complications. Mobile digital health interventions could enhance adherence and reduce cancellations.

**Objective:**

This study assessed the effectiveness of the Sharp Health Companion smartphone app, built on the CareKit health platform, compared with printed instructions. The aim was to evaluate its impact on medication adherence, surgery delays and cancellations, visual outcomes, and patient experience among older adults undergoing cataract surgery.

**Methods:**

In this randomized controlled trial, 200 patients aged 41-87 (mean 69, SD 8.2) years were enrolled at a high-volume ophthalmology practice between December 2022 and January 2024. Most participants (145/200, 73%) were 65 years or older. Patients were randomized to group 1 (printed instructions with phone reminders, n=104) or group 2 (Sharp Health Companion app supplemented with backup printed instructions, n=96). Both groups received identical perioperative instructions and medications. Data included demographics, visual acuity, medication adherence (self-reported by paper checklist or Sharp Health Companion app care-card checklist and eye medication bottle weights), surgery cancellations and delays, and satisfaction surveys administered preoperatively, on postoperative day 1, and at 30 days.

**Results:**

Sharp Health Companion app users had fewer same-day surgery delays (1/96, 1%) than those receiving printed instructions (10/104, 10%; *P*=.01), while cancellation rates were similar (*P*=.33). Patient-reported preparedness for surgery was high and comparable between groups on postoperative day 1 (group 1: mean 9.56, SD 1.19; group 2: 9.77, SD 0.73; *P*=.16). Preparedness for recovery at postoperative month 1 was similarly high (mean 9.92, SD 0.37 vs mean 9.85, SD 0.47; *P*=.28). At 30 days postoperatively, visual acuity improvement was similar (mean 0.14, SD 0.17 vs mean 0.11, SD 0.13 logarithm of the minimum angle of resolution; *P*=.13), and complications were rare (iritis 2/98, 2% vs 1/87,1%; cystoid macular edema 1/98, 1% vs 1/87, 1%). Self-reported medication adherence favored printed instruction users (66/68, 97% vs 47/64, 73%; *P*<.001), whereas objective antibiotic-drop adherence favored app users (mean 5.36, SD 1.17 g vs mean 5.67, SD 1.00 g; *P*=.046).

**Conclusions:**

The Sharp Health Companion app reduced same-day surgery delays and improved patient experience while supporting objective medication adherence in predominantly older adults undergoing cataract surgery. These findings suggest mobile health interventions can enhance perioperative care and efficiency, even in populations less familiar with technology.

**Trial Registration:**

ClinicalTrials.gov NCT07028359; https://clinicaltrials.gov/ct2/show/NCT07028359

## Introduction

### Background

Cataracts are the leading cause of preventable blindness worldwide, affecting millions annually, significantly impacting vision, and placing a considerable strain on health care systems as the global population ages. Cataract surgery, the most commonly performed surgical procedure globally, restores eyesight and significantly improves quality of life, with over 30 million surgeries performed annually [1-3]. At Sharp HealthCare in San Diego, California, over 5000 cataract surgeries are conducted annually. However, rising global demand due to an aging population, combined with surgical backlogs exacerbated by the COVID-19 pandemic, has created substantial barriers to timely care, negatively impacting health care accessibility and patient morbidity [4-6].

Delays in surgery extend beyond administrative issues. Prolonged wait times can result in denser, more complicated cataracts that necessitate longer and technically challenging surgeries, increasing the risk of complications and resource burden [3,7]. Patient adherence to perioperative instructions, both preoperative and postoperative, is vital for optimal outcomes. Missed, forgotten, or poorly understood instructions such as dietary restrictions, medication schedules, self-quarantine requirements, and postoperative care frequently result in surgery delays and compromised surgical outcomes [8,9]. Cancellation rates increased notably during the COVID-19 pandemic, posing financial challenges to health care systems and negatively affecting patient experiences [10-12]. Adherence to postoperative medication regimens, specifically eye drops, is often problematic, potentially leading to complications and suboptimal visual recovery [13-15]. Furthermore, patients undergoing cataract surgery frequently receive intravenous sedation, which may impair memory and hinder recall of postoperative care instructions provided in the recovery room, a challenge further exacerbated by isolation during the COVID-19 pandemic [16,17].

Historically, printed instructions have guided patients through perioperative processes; however, studies indicate inconsistent adherence, with instructions frequently lost or misunderstood, leading to cancellations and adverse outcomes [18,19]. This issue intensified during the COVID-19 pandemic, highlighting significant limitations in the flexibility and responsiveness of printed materials when protocols rapidly changed [20]. Studies have also identified biases among health care providers, who may question older adults’ ability to effectively use digital technology, emphasizing the importance of addressing these perceptions [21]. These barriers underscore the need to better understand the role of mobile digital health interventions to consistently support patients throughout their surgical journey.

### Related Work

**Figure 1:**
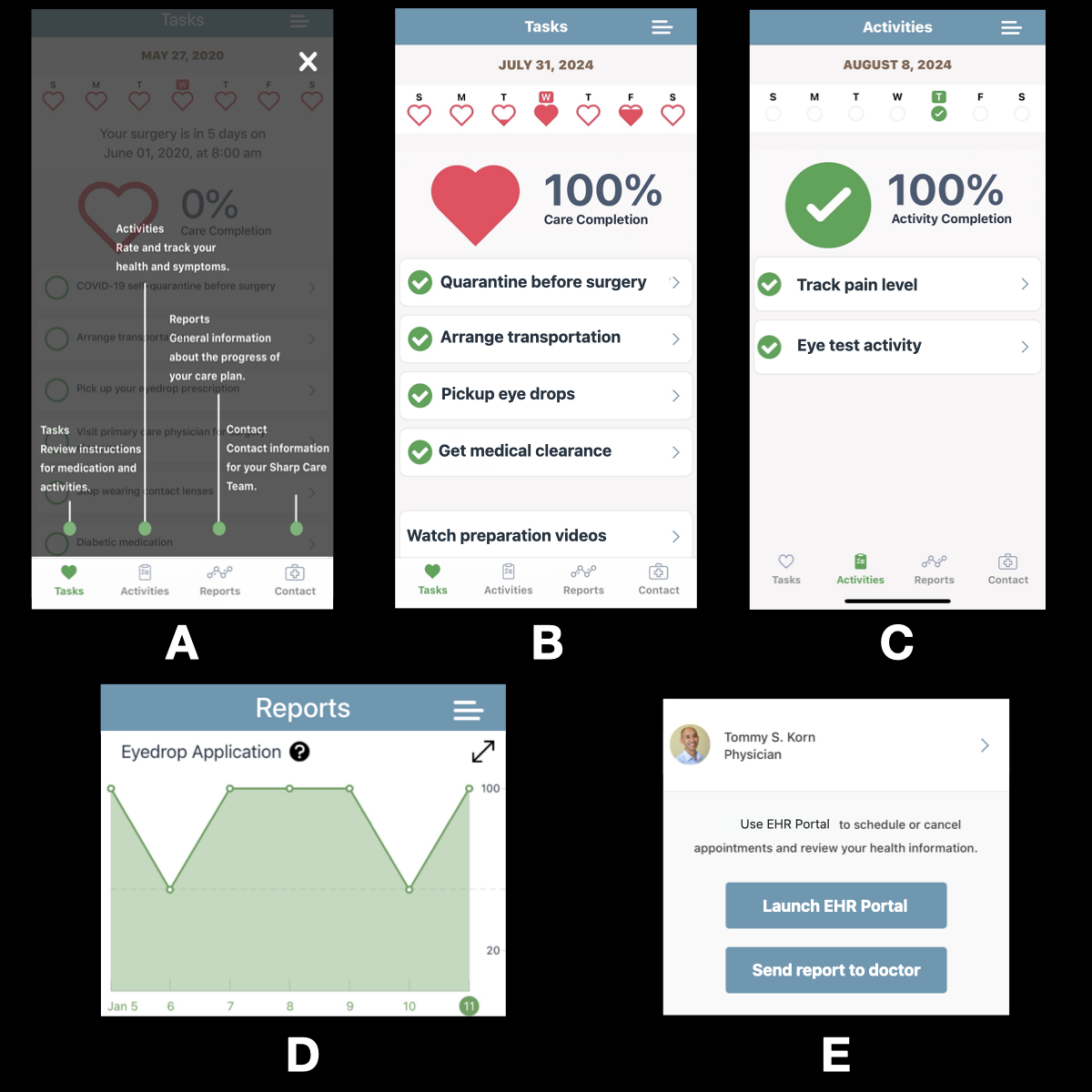
Screenshots of the Sharp Health Companion app illustrating key perioperative features: (A) interactive task overview, (B) care task completion and medication adherence checklist, (C) postoperative symptom tracking (eye pain and visual acuity), (D) patient-reported medication adherence graph, and (E) integrated care team communication portal.

Recognizing the emerging trend toward increasing mobile technology adoption among seniors, Sharp HealthCare proactively explored digital health solutions prior to the COVID-19 pandemic [22]. Introduced in 2016, CareKit is an open-source platform facilitating rapid, cost-effective development of health care–focused mobile apps designed to empower patients in managing their health under clinical guidance [23]. Use of an open-source digital health platform provides critical flexibility for health care systems by creating scalable solutions that effectively address the diverse surgical needs of an ever-increasing geriatric population. Leveraging this opportunity, a multidisciplinary Sharp HealthCare team (ophthalmologists, nurses, developers, and designers) produced and piloted the Sharp Health Companion app in 2017 to support older cataract surgery patients (Figure 1 and Multimedia Appendix 1).

Strategically, the app was initially created as a stand-alone solution that was not integrated directly with the health system’s multiple electronic health record (EHR) platforms, due to an ongoing organizational transition toward a unified EHR system at the time. This approach later provided valuable flexibility, enabling straightforward future integration with the unified EHR. Initial pilot results of the Sharp Health Companion app showed improved medication adherence and reduced surgery cancellations [24].

### Objective

This study examined the role of digital health tools in helping the senior population with their preparation and recovery from cataract surgery. Consistent with systematic reviews indicating that the COVID-19 pandemic accelerated seniors’ adoption of digital health tools and telehealth, this study examined the capability and willingness of older patients to engage with these technologies for remote care [25]. Supported by an administrative portal, the Sharp Health Companion app allowed care teams to instantly disseminate updated surgical care instructions and rapidly evolving COVID-19 safety protocols (self-quarantine and polymerase chain reaction testing reminders) to hundreds of patients simultaneously. This real-time communication and remote monitoring capability ensured the continuity of cataract surgeries at Sharp HealthCare even as elective procedures in other specialties faced significant disruptions.

Previous research has largely focused on digital educational interventions limited to preoperative preparation, lacking comprehensive assessment throughout the full perioperative cataract surgery journey, including the day of surgery and postoperative recovery phases [26,27]. The aim of this randomized controlled trial was to evaluate whether the Sharp Health Companion smartphone app could improve medication adherence and perioperative preparedness compared with traditional paper instructions among predominantly older adults undergoing cataract surgery.

## Methods

### Study Design and Participants

This was a prospective, randomized, masked comparative study conducted over 18 months with the objective of evaluating the effectiveness of a digital health intervention in enhancing perioperative adherence, reducing surgery cancellations, and improving patient experience and outcomes in cataract surgery. Participants were recruited from a high-volume, 2-surgeon cataract ophthalmology practice in a metropolitan setting. This study was reported in accordance with the CONSORT (Consolidated Standards of Reporting Trials) guidelines (Multimedia Appendix 2). Eligible participants were English-speaking adults aged 18 years or older, deemed appropriate surgical candidates for cataract surgery (visual symptoms such as blurred vision and/or daytime or nighttime glare not improved with refraction and prescription eyeglasses or contact lenses, best-corrected visual acuity 20/40 or worse) by their ophthalmologists. Participants were required to verbally confirm basic technical proficiency with their iPhone during the preoperative consultation prior to randomization. Clinic staff informally assessed this by asking whether participants could operate their smartphone, use secure login (eg, Face ID, Touch ID, or passcode), send SMS text messages or email, and access the institution’s EHR portal app. No demonstration was required on the smartphone, ensuring patient privacy and safety. This step ensured that participants had sufficient skills to independently use the study app. Eligibility criteria included participant capacity to understand study requirements, provide informed consent, and authorize the use of their protected health information. Exclusion criteria included visual, auditory, cognitive, or motor impairments that could prevent effective use of the app. Participants with a history of prior cataract surgery were also excluded, as their previous experience would confer an advantage in navigating the perioperative process, potentially biasing the study outcome. No formal a priori power calculation was conducted. The planned target enrollment was approximately 300 participants based on feasibility estimates; however, recruitment of smartphone-capable older adults was challenging.

### Randomization

Eligible participants who elected to undergo cataract surgery and consented to study participation were randomized via a digital randomization app to either group 1 or group 2. Each participant received a unique study identifier to protect personal information and ensure confidentiality. The randomization assignments were masked from the principal investigators, ophthalmologists, and research team, including ophthalmic technicians, administrative staff, and surgery schedulers, who remained masked to group allocation throughout the study.

### Interventions

**Figure 2:**
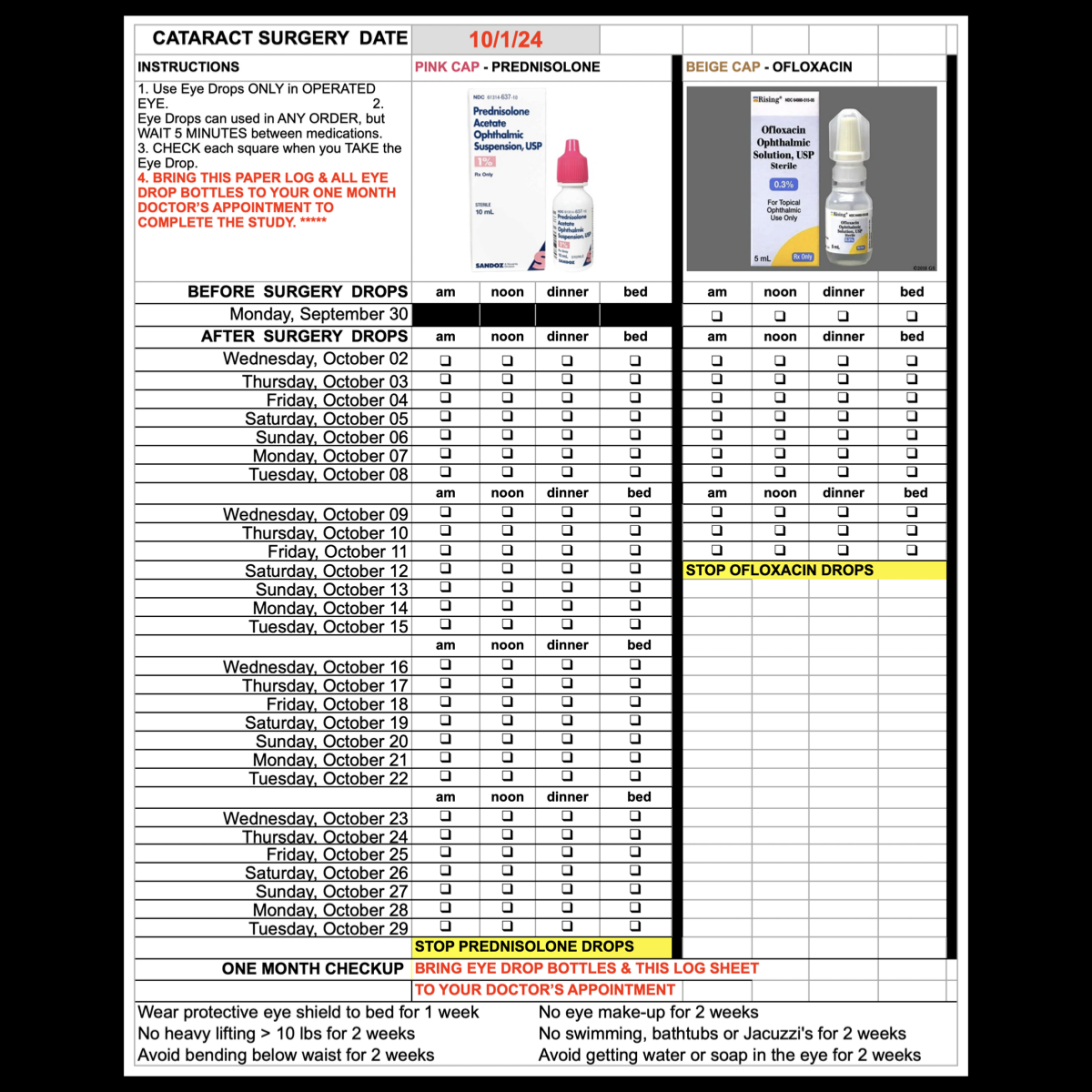
Printed medication adherence checklist provided to group 1 participants using traditional printed instructions, used to track antibiotic and steroid eye drop usage before and after cataract surgery.

Both groups 1 and 2 received the standard printed instructions, and group 2 additionally used the Sharp Health Companion app; the perioperative content was identical across groups, covering preoperative, day of surgery, and postoperative care. Group 1 participants received the standard printed instructions provided at the doctor’s office (Multimedia Appendix 3), which included essential presurgery protocols and a paper-based medication adherence checklist for postoperative use (Figure 2). In addition, group 1 received a 1-time automated telephone reminder the day before surgery. The brief prerecorded message confirmed the scheduled surgery date, time, and check-in location and instructions; it did not include dietary restriction instructions.

**Figure 3:**
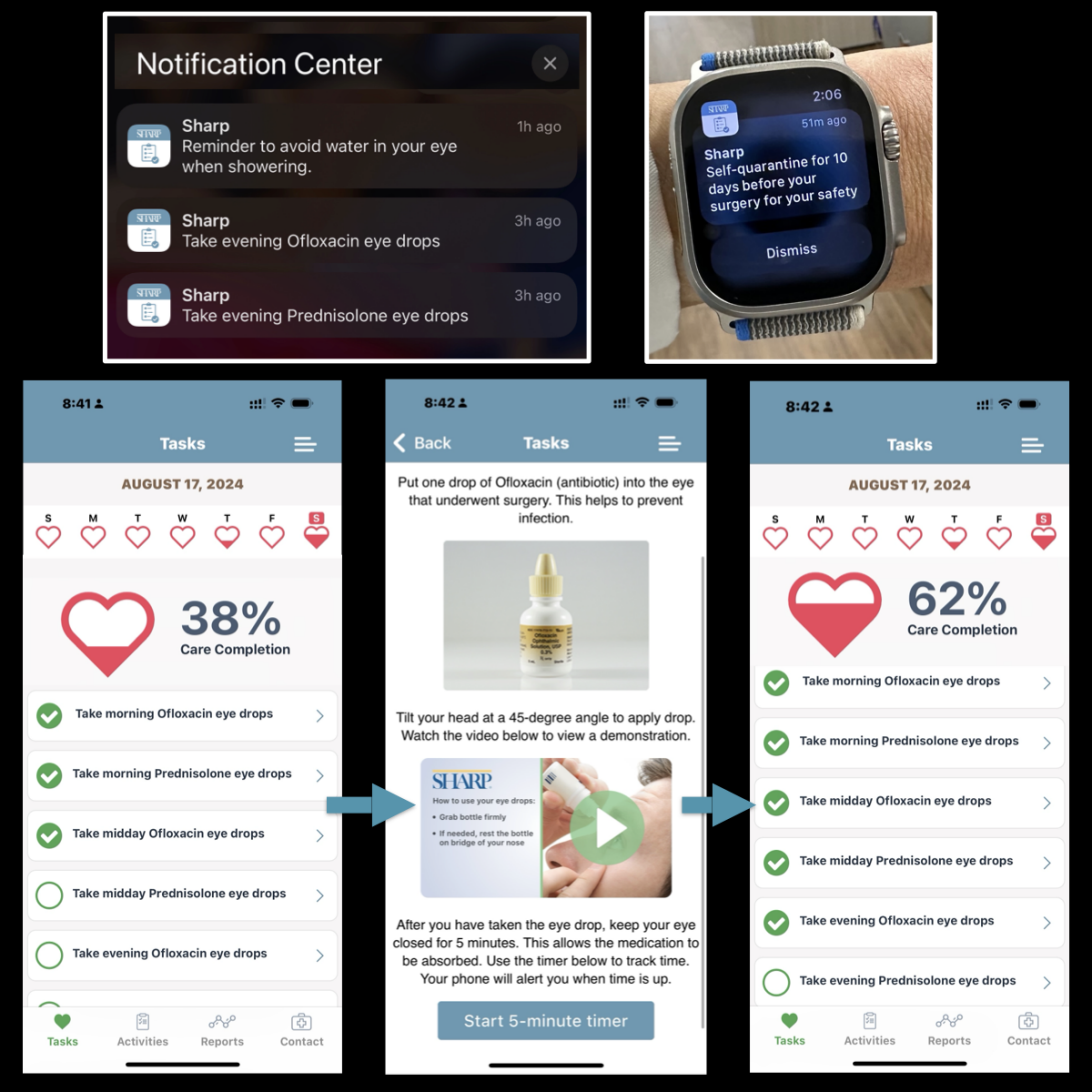
Sharp Health Companion app workflow: smartphone and smartwatch notifications prompt patients to administer eye drops, mark adherence tasks as complete, review instructional videos, and use an integrated 5-minute timer, tracking daily progress toward 100%.

Group 2 participants used the Sharp Health Companion app, developed on the CareKit platform, which provided digital notifications for preoperative requirements such as self-quarantine and polymerase chain reaction testing (during the COVID-19 pandemic), dietary restrictions, surgery appointment details (date, time, and location), and medication reminders. The app featured interactive care checklists, educational videos, and timely eye drop medication adherence reminders delivered via iPhone and/or Apple Watch notifications (Figure 3 and Multimedia Appendix 1). Additionally, the app offered tools for visual acuity self-monitoring, easy access to care team contact information, and postoperative care instructional videos (Figure 1). As a precaution, group 2 also received the same printed instructions (Multimedia Appendix 3) as group 1, serving as a backup in the event of app malfunction or patient technical difficulties. Unlike group 1, participants in group 2 did not receive any automated telephone reminders or a printed medication adherence checklist; the app served as their primary tool for perioperative support, supplemented only by backup printed instructions.

Both groups received identical, standardized eye drop medications at no cost: 1 bottle of prednisolone acetate 1% (10 mL; Sandoz Pharmaceuticals) and 1 bottle of ofloxacin 0.3% (5 mL; Rising Pharmaceuticals), dispensed directly by the institution’s pharmacy to ensure consistency.

### Data Collection

Participant experience was assessed using an adapted version of the Consumer Assessment of Healthcare Providers and Systems-2.0 survey, evaluating clarity of information, preparedness, and overall surgical satisfaction (Multimedia Appendix 4). At their initial cataract consultation, eligible participants from both groups completed the survey to report demographic and technology use characteristics prior to undergoing surgery. Subjective data were collected at three time points: (1) the baseline survey (preoperative office consultation), (2) the postoperative day 1 survey, and (3) the postoperative month 1 survey (Multimedia Appendix 4). Patient confidentiality was ensured by administering surveys on a HIPAA (Health Insurance Portability and Accountability Act)-compliant, enterprise-grade SurveyMonkey platform accessed via secure health system iPads configured in kiosk mode. Participants entered only their assigned study identifier number for the surveys; no personally identifiable information was collected.

Objective medication adherence was determined by weighing antibiotic and steroid eye drop bottles returned at the 30-day postoperative visit. Returned bottles were weighed using an analytical balance (Apollo GX-603A; A&D Weighing), accurate to ±0.001 g, with all exterior packaging removed. To establish a baseline, the average weight of 5 unopened bottles of each medication was recorded: prednisolone acetate 1% (10 mL) weighed 17.223 g, and ofloxacin 0.3% (5 mL) weighed 8.466 g. Each participant was prescribed exactly 1 bottle of each medication for the postoperative course, and all medications were obtained from the same manufacturer to ensure consistency. Bottle weights at 30 days were compared to these baselines to determine the amount of medication consumed. All participants from both groups were instructed to return their bottles to precisely quantify medication usage.

**Figure 4:**
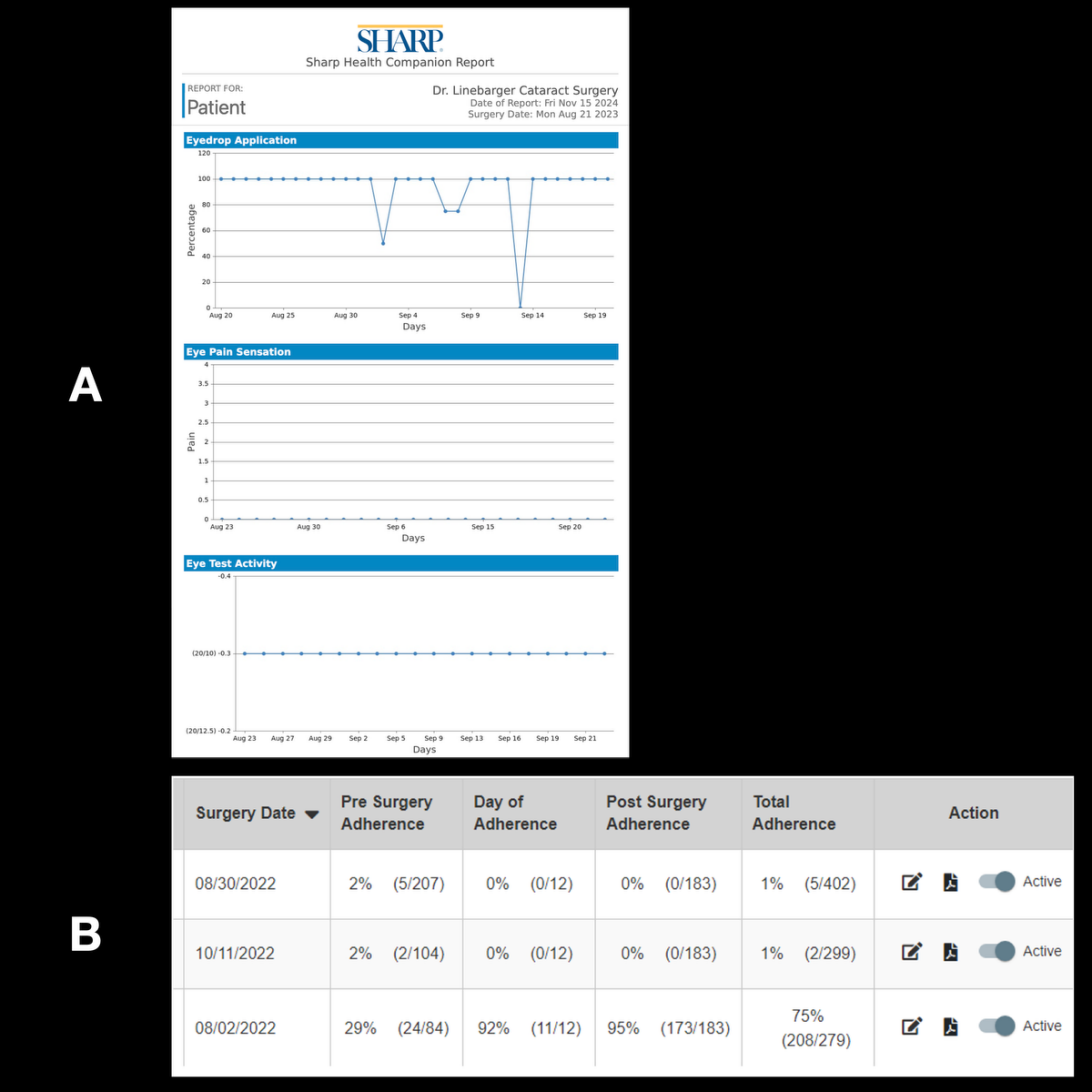
(A) Patient-generated report from the Sharp Health Companion app, showing self-reported medication adherence (eye drops), pain levels, and visual acuity assessments. Patients could review this summary and securely share it with their care team before and after cataract surgery. (B) Care team view from the HIPAA (Health Insurance Portability and Accountability Act)–secure back-end portal, displaying objective eye drop adherence data linked to each participant’s study identifier, enabling clinicians to monitor adherence trends and provide timely support.

In addition, group 1 participants were asked to return their printed medication adherence checklists at the final visit (Figure 2). Group 2 adherence was monitored through a HIPAA-secure, encrypted back-end portal that restricted retrospective reporting beyond 24 hours, ensuring an accurate reflection of daily medication adherence (Figure 4).

**Figure 5:**
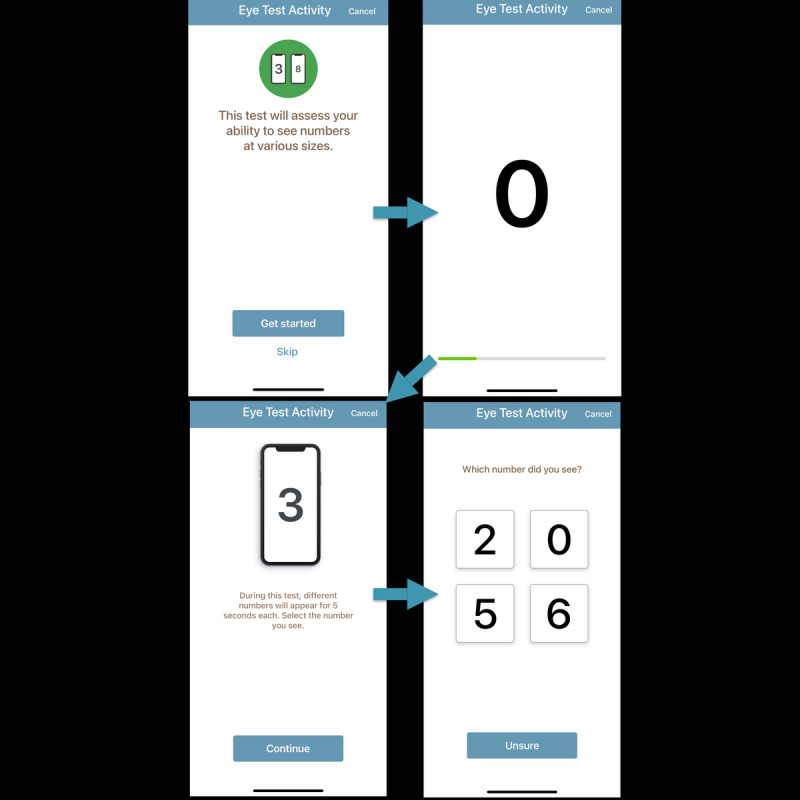
Sharp Health Companion app’s integrated vision self-assessment workflow, guiding patients through instructions and sequential vision testing. The ability to correctly identify smaller numbers indicates better visual acuity (eye performance).

Visual acuity was assessed by ophthalmic technicians using an Early Treatment Diabetic Retinopathy Study chart on the logarithm of the minimum angle of resolution (logMAR) scale. Measurements were recorded preoperatively and at 1 day and 30 days postoperatively, allowing comparison of surgical effectiveness between groups. Additionally, group 2 participants had optional access to a visual acuity self-assessment activity within the Sharp Health Companion app, enabling independent self-monitoring of vision recovery following cataract surgery (Figure 5). The care team could remotely access and review data from this vision activity throughout the postoperative period if participants chose to use this optional feature.

### Outcomes and Measurements

The primary outcomes included patient satisfaction with the care experience, surgery delays and cancellations, and subjective and objective medication adherence. A surgery delay was prespecified as a postponement of >2 hours with the procedure still completed on the scheduled day; a surgery cancellation was defined as a same-day procedure not performed. Reasons were abstracted from the surgery schedule and chart notes by masked staff at the conclusion of the study.

Subjective medication adherence was defined as the number of prescribed eye drop doses marked as completed divided by the total number of prescribed doses. For both groups, antibiotic drops were initiated 1 day before surgery and administered 4 times daily (4 doses preoperatively), then continued at the same frequency for 10 days postoperatively (40 doses in total). Steroid drops were initiated on the day of surgery and taken 4 times daily for 30 days (120 total doses). Group 1 participants manually checked off each completed dose on printed checklists (Figure 2), while group 2 participants recorded each administered dose digitally within the Sharp Health Companion app (Figures 3 and 4B). The app required daily reporting and did not allow retrospective entries beyond 24 hours, capturing real-time adherence data.

Study completion was a composite outcome that included cataract surgery performed, attendance at all scheduled postoperative visits, completion of all 3 surveys, and return of both antibiotic and steroid bottles at 30 days. In addition, group 1 participants were required to return the printed medication adherence checklist, while group 2 participants’ medication adherence was verified through the Sharp Health Companion app portal monitored by the research team (Figure 4). Secondary outcomes encompassed postoperative visual acuity and postsurgical complications, such as cystoid macular edema and uveitis at the final 30-day appointment after cataract surgery. Postoperative iritis was determined by slit-lamp examination, documenting anterior chamber cells/flare requiring treatment by the ophthalmologist. Cystoid macular edema was diagnosed clinically and confirmed with optical coherence tomography when indicated. The likelihood of recommending the health care organization item was measured on a 1-10 scale (1=not at all likely to 10=extremely likely).

### Statistical Analysis

Statistical analyses were conducted using SPSS Statistics for Mac (version 31.0; IBM Corp). Continuous outcomes were compared with independent *t* tests, and categorical outcomes with chi-square tests. All analyses adhered to the intention-to-treat principle, with participants analyzed in the groups to which they were randomized, regardless of adherence or protocol deviations. Statistical significance was set at *P*<.05.

### Ethical Considerations

This study was approved by the Sharp HealthCare Institutional Review Board (approval number 2209803) and adhered to institutional guidelines and the principles of the Declaration of Helsinki. All participants provided informed consent via DocuSign and received a digital and/or printed copy of the California Experimental Subject’s Bill of Rights. Participants were informed of their ability to withdraw at any time during the study without affecting their clinical care. To protect privacy, participants were assigned unique identifiers, surveys were administered via HIPAA-compliant software (SurveyMonkey Enterprise, kiosk mode), and app data were encrypted on secure servers. Participants did not receive financial compensation. Prescribed eye medications (prednisolone acetate 1% and ofloxacin 0.3%) were provided at no cost as part of study participation.

## Results

### Participant Demographics

**Figure 6:**
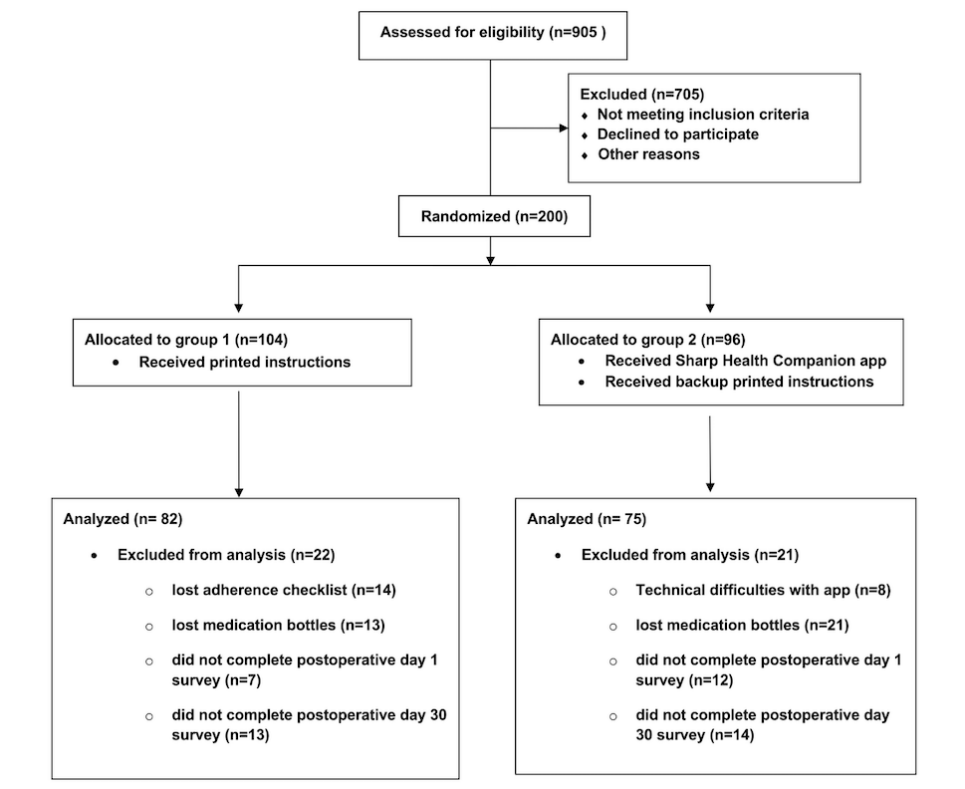
CONSORT (Consolidated Standards of Reporting Trials) flow diagram. Flow of participants through the randomized controlled trial, including numbers assessed for eligibility, excluded, randomized, allocated to group 1 (printed instructions) or group 2 (Sharp Health Companion app), and included in the final analyses. Some participants met more than 1 exclusion criterion (eg, lost both the adherence checklist and medication bottles or experienced technical difficulties and lost bottles).

Between December 2022 and January 2024, a total of 905 patients were referred for cataract surgery, and 200 were enrolled and randomized into 2 groups: group 1 (printed instruction participants; n=104) and group 2 (Sharp Health Companion app participants; n=96; Figure 6). Participants ranged in age from 41 to 87 years, with an overall mean age of 69 (SD 8.2) years. The app group was slightly younger than the printed instruction group (mean 67.1, SD 8.7 years vs mean 69.9, SD 7.8 years; *P*=.02). Most participants were aged ≥65 years (145/200, 73%), with no difference between groups (*P*=.25). The majority were female (140/200, 70%), and gender distribution did not differ between groups (*P*=.50). Racial and ethnic distributions were also comparable (*P*=.39). Nearly all participants reported Wi-Fi access (185/200, 93%), slightly higher in the printed instruction group (100/104, 96% vs 85/96, 89%; *P*=.04). Comfort with smartphone apps (1=least comfortable to 5=most comfortable) was higher among app participants (mean 3.67, SD 0.68 vs mean 3.36, SD 0.82; *P*<.001). Smartwatch ownership was more common among app users (55/96, 57% vs 38/104, 37%; *P*=.003). Prior experience with smartphone EHR portal apps was similar between groups (*P*=.15; Table 1).

**Table 1:** Demographics of study participants (n=200).

Characteristic		Group 1 (printed instructions; n=104)	Group 2 (Sharp Health Companion app; n=96)	*P* value	
Age (years), mean (SD)		69.9 (7.8)	67.1 (8.7)	.02^a,b^	
Age (≥65 years), n (%)		79 (76)	66 (69)	.25^c^	
**Gender, n (%)**					.50^c^
	Male	29 (28)	31 (32)		
	Female	75 (72)	65 (68)		
**Ethnicity, n (%)**					.39^c^
	White	78 (75)	71 (74)		
	Hispanic/Latino	13 (12)	7 (7)		
	Asian or Pacific Islander	8 (8)	12 (13)		
	Black or African American	2 (2)	4 (4)		
	American Indian or Alaskan Native	0 (0)	1 (1)		
	Other	2 (2)	0 (0)		
	Prefer not to answer	1 (1)	1 (1)		
**Primary home internet connection, n (%)**					
	Wi-Fi	100 (96)	85 (89)	.04^b,c^	
	Mobile cellular plan	4 (4)	11 (11)	.07^d^	
Comfort with smartphone apps (1=least comfortable to 5=most comfortable), mean (SD)		3.36 (0.82)	3.67 (0.68)	<.001^a,b^	
Prior use of smartphone medical apps (eg, EHR^e^ portal apps), n (%)		78 (75)	80 (83)	.15^c^	
Uses smartwatch (Apple Watch, Fitbit, etc), n (%)		38 (37)	55 (57)	.003^b,c^	

^a^Independent *t* test (2-tailed).

^b^Statistically significant (*P*<.05).

^c^Chi-square test.

^d^Fisher exact test.

^e^EHR: electronic health record.

### Preparedness and Clarity of Instructions: Postoperative Day 1 Survey

Participants completed an in-office survey 1 day after cataract surgery to assess preparedness, clarity, and usability of preoperative instructions (Table 2 and Multimedia Appendix 4). Most participants reviewed their instructions within 2-7 days before surgery (100/181, 55%), with no significant difference in timing between groups (*P*=.21). Among app participants, 56% (47/84) used both the Sharp Health Companion app and backup printed instructions before surgery.

**Table 2:** Patient-reported preparedness and clarity of preoperative instructions (postoperative day 1 survey; n=181).

Preparedness for surgery		Group 1 (printed instructions; n=97)^a^		Group 2 (Sharp Health Companion app; n=84)^a^		*P* value	
**Q10: Before your surgery, what did you use to help prepare for surgery, n (%)**							—^b^
	Printed instructions	97 (100)		10 (12)			
	App	N/A^c^		27 (32)			
	App + printed instructions backup	N/A		47 (56)			
**Q11: When did you review your information (paper instructions and/or Sharp Health Companion app) before surgery, n (%)**							.21^d^
	Over 31 days before surgery	6 (6)		5 (6)			
	8-30 days before surgery	16 (15)		26 (31)			
	2-7 days before surgery	59 (57)		41 (49)			
	1 day before surgery	13 (13)		9 (11)			
	On the day of surgery	2 (2)		3 (4)			
**Q12: Before your surgery, did anyone in this surgeon’s office give you all the information you needed about your eye surgery, n (%)**							.63^d^
	Yes, definitely	88 (92)		80 (95)			
	Yes, somewhat		6 (6)		3 (4)		
	No	2 (2)		1 (1)			
**Q13: Before your surgery, did anyone in this surgeon’s office give you easy to understand instructions about getting ready for your eye surgery, n (%)**							.02^d,e^
	Yes, definitely	86 (90)		83 (99)			
	Yes, somewhat	8 (8)		0 (0)			
	No	2 (2)		1 (1)			
Q14: Before your surgery, on a scale from 1 to 10, how would you rate your preparedness for surgery given the information you received from the surgeon’s office (1-10 scale, 1=least prepared, 10=most prepared), mean (SD)		9.56 (1.19)		9.77 (0.73)		.16^f^	
Q15: How would you rate your surgery experience so far with the information you’ve been provided (1-10 scale, 1=least favorable, 10=most favorable), mean (SD)		9.92 (0.37)		9.96 (0.24)		.32^f^	
**Q16: If you used the Sharp Health Companion app, did you have to use the backup paper instructions at any time, n (%)**							—
	Yes, definitely used the paper instructions	N/A		16 (19)			
	Yes, somewhat used the paper instructions	N/A		37 (44)			
	No, I did not have to use my backup paper instructions	N/A		31 (37)			
**Q17: How does the Sharp Health Companion app compare to other medical apps you have used, n (%)**							—
	Easier and more valuable	N/A		44 (54)			
	About as easy and valuable	N/A		37 (46)			
	Not as easy or valuable	N/A		0 (0)			
**Q18: I would use the Sharp Health Companion app for other surgeries or medical procedures if available, n (%)**							—
	Yes, definitely	N/A		68 (87)			
	Yes, somewhat	N/A		10 (13)			
	No	N/A		0 (0)			
Patients who initiated communication (phone calls or emails) with staff regarding surgery logistics, n (%)		9 (9)		6 (7)		.62^d^	

^a^Sample sizes differ from the total group because not all participants completed this survey item.

^b^Not available.

^c^N/A indicates response options that were not applicable to a group (eg, group 1 did not have access to the Sharp Health Companion app).

^d^Pearson chi-square test.

^e^Statistically significant (*P*<.05).

^f^Independent *t* test.

Regarding clarity of instructions, response distributions (“yes, definitely,” “yes, somewhat,” or “no”) did not differ significantly between groups (*P*=.63). Most participants rated their instructions as “definitely easy to understand,” with slightly higher ratings among app users compared with printed instruction users (83/84, 99% vs 86/96, 90%; *P*=.02). Preparedness ratings on a 1-10 scale (1=least prepared to 10=most prepared) were high and similar in both groups (mean 9.56, SD 1.19 vs mean 9.77, SD 0.73). Surgery experience ratings were likewise high (*P*=.32).

Among app users, 63% (53/84) reported using backup printed instructions to some extent, while the minority 37% (31/84) did not need them. When comparing the Sharp Health Companion app to other medical apps, 54% (44/84) rated it as easier and more valuable, and 46% (37/84) as about as easy and valuable; none rated it as less valuable. Most app participants (68/84, 87%) indicated they would “definitely” use the Sharp Health Companion app for future medical or surgical procedures, and the remainder (10/84, 13%) responded, “yes, somewhat.” Finally, a small number of participants contacted the surgeon’s office for clarification about surgery logistics, with no significant difference between groups (9/97, 9% vs 6/84, 7%; *P*=.62).

### Cataract Surgery Delays and Cancellations

Surgery delays were significantly more frequent among participants receiving printed instructions than those using the Sharp Health Companion app (10/104, 10% vs 1/96, 1%; *P*=.01). Delays were defined as procedures postponed by >2 hours but completed on the scheduled day, most commonly due to participants arriving at the incorrect surgery location or time. Surgery cancellations occurred in 15 participants overall, with similar proportions in the printed instruction and app groups (6/104, 6% vs 9/96, 9%; *P*=.33). The primary reasons included acute illness, participant-initiated withdrawal, and financial or insurance issues (Table 3).

**Table 3:** Cataract surgery delays and cancellations (n=200).

Outcome measure			Group 1 (printed instructions; n=104), n (%)		Group 2 (Sharp Health Companion app; n=96), n (%)		*P* value
**Delayed surgery**			10 (10)		1 (1)		.01^a,b^
	Missed dietary restrictions	2 (2)		0 (0)			
	Wrong location/time	6 (6)		1 (1)			
	Other reasons	2 (2)		0 (0)			
**Canceled surgery**			6 (6)		9 (9)		.33^c^
	Illness	3 (3)		2 (2)			
	Missed dietary restrictions	1 (1)		0 (0)			
	Wrong location/time	1 (1)		0 (0)			
	Self-canceled	1 (1)		5 (5)			
	Insurance/financial reasons	0 (0)		1 (1)			
	Not a candidate	0 (0)		1 (1)			

^a^Statistically significant (*P*<.05).

^b^Fisher exact test.

^c^Chi-square test.

### Study Completion and Adherence to Protocol

Study completion rates were high and comparable between groups (82/104, 79% vs 75/96, 78%). Among app users, engagement with specific features varied. Most participants used both the Sharp Health Companion app and backup printed instructions (42/75, 56%), while smaller subsets used the app alone (24/75, 32%) or printed instructions only (9/75, 12%). About half completed the vision self-assessment (38/75, 51%) and eye pain self-assessment (37/75, 49%) activities within the app. Loss of medication bottles was slightly higher in the app group (21/75, 28%) compared to the printed instruction group (13/82, 16%) but the difference was not statistically significant (*P*=.07). Some participants (8/75, 11%) in the app group reported technical issues, such as forgotten login credentials or intermittent software errors that interfered with adherence tracking. Overall, adherence and study completion were comparable between groups, indicating that the addition of the Sharp Health Companion app did not adversely affect protocol compliance (Table 4).

**Table 4:** Study completion and adherence to protocol (n=200).

Study completion details			Group 1 (printed instructions; n=104), n (%)		Group 2 (Sharp Health Companion app; n=96), n (%)		*P* value
**Participants who fully completed the study**			82 (79)		75 (78)		.90^a^
	Used backup printed instructions only	N/A^b^		9 (12)			
	Used the Sharp Health Companion app only	N/A		24 (32)			
	Used the Sharp Health Companion app and backup printed instructions	N/A		42 (56)			
	Used the vision self-assessment test activity in the app	N/A		38 (51)			
	Used the eye pain self-assessment activity in the app	N/A		37 (49)			
**Participants with incomplete data who were excluded from the analysis**			22 (27)		21 (28)		
	Lost printed medication adherence checklist (Figure 2)	14 (17)		N/A		.90^a^	
	Lost medication bottles	13 (16)		21(28)		.07^a^	
	Technical difficulties with the app	N/A		8 (11)		.90^a^	

^a^Chi-square test.

^b^N/A indicates response options that were not applicable to a group (eg, group 1 did not have access to the Sharp Health Companion app).

### Preparedness and Clarity of Instructions: Postoperative Month 1 Survey

At the month 1 follow-up visit, nearly all participants in both groups reported receiving complete and clear preoperative information (Table 5 and Multimedia Appendix 4). Almost all participants indicated that someone in the surgeon’s office provided all the information they needed about their eye surgery, and nearly all rated the preoperative instructions as easy to understand. Mean preparedness ratings before and after surgery were uniformly high and comparable between groups, and no statistically significant differences were noted. All participants in both groups reported clear recovery instructions, and overall surgery experience ratings remained similar. Among app users, the majority (49/82, 60%) relied solely on the Sharp Health Companion app, while the remainder also used backup printed instructions. Most app users rated the app as easier or more valuable than other medical apps (49/82, 60%) and nearly all indicated they would use a similar app again for other surgeries or medical procedures (78/82, 95%). Participants from both groups rated their likelihood of recommending Sharp HealthCare to a friend or colleague based on their experience highly (mean 9.83, SD 0.59 vs mean 9.74, SD 0.91), with no significant difference between groups (*P*=.43).

**Table 5:** Patient-reported preparedness and clarity of preoperative instructions: postoperative month 1 survey (n=174).

Outcome measure		Group 1 (printed instructions; n=92)^a^		Group 2 (Sharp Health Companion app; n=82)^a^		*P* value		
**Q20: Before your surgery, did anyone in this surgeon’s office give you all the information you needed about your eye surgery, n (%)**							.94^b^	
	Yes, definitely	90 (99)		81 (99)				
	Yes, somewhat	1 (1)		1 (1)				
	No	0 (0)		0 (0)				
**Q21: Before your surgery, did anyone in this surgeon’s office give you easy to understand instructions about getting ready for your eye surgery, n (%)**							.34^b^	
	Yes, definitely	91 (99)		82 (100)				
	Yes, somewhat	1 (1)		0 (0)				
	No	0 (0)		0 (0)				
Q22: Before your surgery, on a scale from 1 to 10, how would you rate your preparedness for surgery given the information you received from the surgeon’s office, (1-10 scale: 1=least prepared to 10=most prepared), mean (SD)		9.86 (0.38)		9.88 (0.40)		.74^c^		
Q23: After your surgery, on a scale from 1 to 10, how would you rate your preparedness for surgery given the information you received from the surgeon’s office, (1-10 scale: 1=least prepared to 10=most prepared), mean (SD)		9.92 (0.37)		9.85 (0.47)		.28^c^		
**Q24: Did anyone in this surgeon’s office give you easy to understand instructions about what to do during your recovery period?, n (%)**								—^d^
	Yes, definitely		92 (100)		82 (100)			
	Yes, somewhat	0 (0)		0 (0)				
	No	0 (0)		0 (0)				
Q25: How would you rate your overall surgery experience from preparation to recovery using the information you were provided (1-10 scale: 1=least favorable to 10=most favorable), mean (SD)		9.92 (0.45)		9.78 (1.04)		.23^c^		
**Q26: If you used the Sharp Health Companion app, did you have to use the backup paper instructions at any time, n (%)**								—
	Yes, definitely used the paper instructions	N/A^e^		6 (7)				
	Yes, somewhat used the paper instructions	N/A		27 (33)				
	No, I did not have to use my backup paper instructions	N/A		49 (60)				
**Q27: How does the Sharp Health Companion app compare to other medical apps you have used, n (%)**								—
	Easier and more valuable	N/A		49 (60)				
	About as easy and valuable	N/A		27 (33)				
	Not as easy or valuable	N/A		3 (4)				
**Q28: I would use the Sharp Health Companion app for other surgeries or medical procedures if available, n (%)**								—
	Yes, definitely	N/A		72 (88)				
	Yes, somewhat	N/A		6 (7)				
	No	N/A		1 (1)				
Q29: How likely is it that you would recommend Sharp HealthCare to a friend or colleague based on your experience (1-10 scale: 1=no at all likely to 10=extremely likely), mean (SD)		9.83 (0.59)		9.74 (0.91)		.43^c^		

^a^Sample sizes differ from the total group n because not all participants completed this survey item.

^b^Pearson chi-square test.

^c^Independent *t* test.

^d^Not available.

^e^N/A indicates response options that were not applicable to a group (eg, group 1 did not have access to the Sharp Health Companion app).

### Clinical Outcomes Following Cataract Surgery: Month 1 Visit

Clinical outcome data were available for 185 participants, including 98 in the printed instruction group and 87 in the app group. This accounted for 15 participants whose surgeries were canceled (6 in the printed instruction group and 9 in the app group). At the month 1 postoperative visit, mean visual acuity (logMAR) was comparable between the printed instruction and app groups (mean 0.14, SD 0.17 vs mean 0.11, SD 0.13; *P*=.13; Table 6). Postoperative inflammation and macular edema were uncommon, with no significant differences noted. Iritis occurred in 2/98 (2%) in the printed instruction group and 1/87 (1%) in the app group, and cystoid macular edema occurred in 1/98 (1%) and 1/87 (1%), respectively. No other postoperative complications were identified.

**Table 6:** Clinical outcomes following cataract surgery: month 1 visit (n=185).

Outcome Measure	Group 1 (printed instructions; n=98)	Group 2 (Sharp Health Companion app; n=87)	*P* value
Visual acuity (ETDRS^a^; logMAR^b^), mean (SD)	0.14 (0.17)	0.11 (0.13)	.13^c^
Postoperative iritis incidence, n (%)	2 (2)	1 (1)	>.99^d^
Postoperative cystoid macular edema incidence, n (%)	1 (1)	1 (1)	>.99^d^

^a^ETDRS: Early Treatment Diabetic Retinopathy Study.

^b^logMAR: logarithm of the minimum angle of resolution.

^c^Independent *t* test.

^d^Fisher exact test.

### Eye Medication Adherence: Patient-Reported vs Objective Measures

Medication adherence data were available for 132 participants, including 68 in the printed instruction group and 64 in the app group. This total reflects participants who returned their medication bottles and completed adherence documentation; others were excluded because printed checklists were misplaced or bottles were lost or not returned at the end of the study.

Self-reported medication adherence was significantly higher among participants using printed checklists than those using the app (66/68, 97% vs 47/64, 73%; *P*<.001; Table 7). Objective medication adherence, assessed by returned eye medication bottle weights, showed broadly similar findings between groups. Residual antibiotic bottle weights were higher in the printed instruction group (mean 5.67, SD 1.00 g vs mean 5.36, SD 1.17 g; *P*=.046), while steroid bottle weights were comparable (mean 11.80, SD 2.00 g vs mean 11.39, SD 1.95 g; *P*=.14).

**Table 7:** Eye medication adherence: self-reported vs objective measures (n=132).

Measure	Group 1 (printed instructions; n=68)	Group 2 (Sharp Health Companion app; n=64)	*P* value
Self-reported eye drop medication adherence, mean (%)^a^	97	73	<.001^b,c^
Final antibiotic bottle weight (g), mean (SD)	5.67 (1.00)	5.36 (1.17)	.05^a,b^
Final steroid bottle weight (g), mean (SD)	11.80 (2.00)	11.39 (1.95)	.14^a^

^a^Adherence represents the mean percentage of prescribed doses completed per participant (161 total doses per patient in both groups).

^b^Independent *t* test.

^c^Statistically significant (*P*<.05).

## Discussion

### Principal Findings

This randomized controlled trial found that use of the Sharp Health Companion smartphone app improved medication adherence, increased patient confidence, and reduced cataract surgery delays compared with printed instructions.

This study uniquely evaluated how a perioperative app during the COVID-19 pandemic could help predominantly older patients receive seamless care, highlighting digital health’s potential to sustain patient engagement during evolving health care conditions. A key finding was the significantly lower rate of surgery delays in the app group 2 compared with the printed group 1, suggesting improved patient readiness and adherence to preoperative instructions. Patient noncompliance with preoperative guidelines, such as dietary restrictions, commonly leads to surgery cancellations and delays, incurring significant hospital and societal costs [12]. The app’s automated reminders and checklists likely mitigated these oversights, aligning with previous findings from a spine surgery mobile app study where app interventions reduced delays and cancellations [28].

Similar postoperative visual acuity improvements were observed in both groups, underscoring that both digital and printed instruction–based methods can support successful cataract surgery outcomes. The comparable, low rates of complications (postoperative iritis and cystoid macular edema) in both groups suggest that the app did not increase the risk of adverse outcomes. These data support using an app alongside printed instructions in routine cataract surgery care.

### Comparison With Prior Work

This study is among the first randomized, controlled trials evaluating a smartphone app vs standard printed instructions specifically in ophthalmology and cataract surgery. A recent European study also explored an app-based perioperative approach with cataract surgery but focused on the app’s user-friendliness and patient acceptance of digital education [27]. In contrast, this study comprehensively evaluated the full perioperative experience of predominantly older adults undergoing cataract surgery, incorporating subjective patient experiences, objective adherence measures, clinical outcomes, and relevant health system metrics.

Prior studies across various surgical domains, including neurosurgery, general surgery, and orthopedic surgery, have consistently demonstrated improvements in adherence, patient satisfaction, and reductions in cancellations and unplanned visits with digital interventions [29,30]. This study’s focused approach on older patients undergoing cataract surgery addresses a critical gap in digital health research, complementing broader telehealth adoption trends during the COVID-19 pandemic [31].

### Adherence and Patient Behavior

We observed notable differences in medication adherence: participants using printed checklists reported higher adherence compared with app users, yet objective measures (eye drop bottle weights) indicated superior adherence among app users. This discrepancy likely reflects a combination of factors. Patients often overreport adherence on printed checklists due to social desirability and recall biases [32], whereas the app required real-time tracking and prevented retrospective entries, potentially capturing a more accurate representation of daily use. Bottle weights also reflect unavoidable variability, such as wastage from repeated instillation attempts, differences in patient technique, or clinical variation in dosing intensity (eg, additional drops prescribed for postoperative inflammation). Multiple studies in ophthalmology and other surgical disciplines have similarly demonstrated that self-reported adherence overestimates true use compared with objective electronic monitoring [33-35]. Importantly, improved objective adherence did not negatively affect clinical outcomes, as visual acuity improvements and postoperative complication rates were comparable between groups, confirming the effectiveness and safety of digital perioperative guidance.

### Patient Experience and Acceptability

Patient experience is a critical aspect of perioperative care, and the Sharp Health Companion app received overwhelmingly positive feedback from participants. Despite advanced age, many app users reported ease of use, comfort after initial onboarding, and appreciation for automatic reminders on eye drops and fasting. Many participants noted feeling calmer and more prepared, highlighting the app’s psychological benefits. Our findings align with broader evidence from similar studies where predominantly older adults demonstrated high acceptance and usability of perioperative apps [36]. Importantly, tailored digital tools have shown value for patients with limited health literacy, providing standardized, clear instructions that may surpass traditional printed instruction methods in effectiveness [37-39].

### Limitations

This study had several limitations. The sample was limited to English-speaking iPhone users. Although an Android version of the Sharp Health Companion app was initially developed, extensive software and hardware fragmentation among Android devices resulted in persistent technical issues and inconsistent user experiences. To ensure a uniform and reliable user experience and accurately address the study objectives, we chose to exclusively enroll iPhone users in this prospective study. Limiting the study to English speakers may also restrict generalizability, and future research should test multilingual app versions to improve accessibility in more diverse populations. Consequently, this potentially limited the generalizability of our findings across diverse patient populations and other smartphone platforms. Additionally, this study was conducted at a single, high-volume urban ophthalmology practice, and our findings may not reflect experiences in rural or lower-income settings that can affect mobile health adoption [40].

There was an unexpected statistically significant difference in participants’ age, comfort with smartphone apps between groups, and usage of smartwatches, raising the possibility of inadvertent selection bias or unequal participant enthusiasm for digital technology despite randomization. Future studies could address this limitation through stratified randomization based on participants’ baseline technology comfort.

Additionally, significantly more participants in group 2 regularly used smartwatches, potentially providing an inherent advantage through increased familiarity with digital notifications and convenient adherence prompts that may have influenced study outcomes. Additionally, group 2 (app users) received backup printed instructions for safety reasons, potentially conferring an advantage by providing supplementary resources unavailable to group 1 participants.

The predominance of female participants, although consistent with broader trends in digital health research among predominantly older adults reflecting higher health care engagement among women, may limit the generalizability of results to a broader population [41].

The study excluded patients with prior cataract surgeries, focusing only on first-time cataract surgery patients. This control helped standardize patient experience and outcomes, but may limit the generalizability of findings to patients with previous surgical experience. All participants were provided with free eye medications, potentially influencing adherence rates positively compared with typical real-world experiences where medication costs could impact adherence.

A notable proportion of participants in both groups did not complete all study procedures, resulting in missing adherence data due to lost medication bottles or incomplete postoperative surveys from missed follow-up appointments. This finding is consistent with inherent challenges noted in previous clinical studies involving predominantly older adults, who may experience difficulties with medication management, memory, or adherence documentation [39].

Furthermore, subjective survey responses regarding patient experience and satisfaction are vulnerable to social desirability and recall bias, potentially leading to inflated satisfaction or adherence reporting. Although we used deidentified objective adherence measures (medication bottle weights), the complete elimination of subjective bias in self-report metrics was not feasible.

Finally, the follow-up duration of this study was limited to immediate postoperative outcomes (up to 30 days), and we did not evaluate long-term medication adherence or sustained engagement with the app beyond the initial postoperative period. Future research should include longer-term assessments to fully capture sustained benefits and adherence behaviors.

### Conclusions and Future Directions

Cataract surgery is the most commonly performed surgical procedure globally. With an aging population, demand for this essential vision-restoring surgery continues to rise. This study demonstrates that the Sharp Health Companion app is a feasible, acceptable, and effective mobile digital tool to support patients, predominantly older adults, throughout their cataract surgery journey. As one of the first randomized controlled trials in ophthalmology directly comparing a digital health intervention against traditional printed instructions, it contributes robust evidence that digital platforms can safely and effectively enhance geriatric surgical care. Specifically, our study suggests the role of digital apps in geriatric surgical care may help reduce delays, increase medication adherence, and enhance patient experience.

Leveraging the CareKit open-source platform enabled rapid, cost-effective development of the Sharp Health Companion app, progressing from concept to minimum-viable product in just 8 months, substantially quicker than typical app development cycles. This accelerated timeline proved especially beneficial when the COVID-19 pandemic unexpectedly emerged, allowing our institution to swiftly adapt perioperative care protocols and continue elective cataract surgeries even as other elective procedures faced significant disruption. Our study also demonstrates older patients’ capability and willingness to adopt digital health tools, emphasizing the importance of agile digital health tools in rapidly evolving and challenging health care scenarios.

Our findings suggest that perioperative app companions for older adults could soon become standard tools across various surgical disciplines. Future research should expand these digital care apps to broader populations supporting multiple languages and explore integration with advanced technologies, including artificial intelligence, spatial computing, and cost-effective electronic medication monitoring. Patient-centered digital solutions hold promise for optimizing outcomes, enhancing care experiences, supporting clinician efficiency, and improving overall health system performance.

## Data Availability

The datasets generated and analyzed during this study are not publicly available due to patient privacy and Health Insurance Portability and Accountability Act regulations. Deidentified summary data supporting the findings of this study are available from the corresponding author upon reasonable request with approval by the Sharp HealthCare Institutional Review Board.

## Appendix

Multimedia Appendix 1 Demonstration video of the Sharp Health Companion CareKit app.

Multimedia Appendix 2 CONSORT-eHEALTH checklist (V 1.6.1).

Multimedia Appendix 3 Backup printed instructions with perioperative logistics for cataract surgery for both study groups.

Multimedia Appendix 4 Complete list of patient survey questions administered at three time points: baseline (preoperative office consultation), postoperative day 1, and postoperative month 1.

## Abbreviations

CONSORT: Consolidated Standards of Reporting Trials
EHR: electronic health record
HIPAA: Health Insurance Portability and Accountability Act
logMAR: logarithm of the minimum angle of resolution
