# Supplementary material for: Digital Health Support for Cataract Surgery With the Sharp Health Companion CareKit App: Randomized Controlled Trial
Source: JMIR Mhealth Uhealth. 2026 Apr 29;14:e78710. doi: 10.2196/78710 (PMC13173071; doi:10.2196/78710)
Supplement: Multimedia Appendix 3 [file mhealth_v14i1e78710_app3.pdf]

Cataract Surgery Checklist

Name: **Eye:**

Surgery Date: ____________________

WITHIN 30 DAYS BEFORE SURGERY:

See your primary care doctor for medical clearance within 30 days BEFORE eye surgery date. If medical clearance is NOT received, your eye surgery will be cancelled for your safety.

WITHIN 10 DAYS BEFORE SURGERY:

Please self-quarantine at home 10 days before your surgery to protect yourself & your care team from COVID-19 infection. This reduces the risk of any vision or life-threatening complications & helps lead to a successful recovery after your surgery.

Please seek medical attention if you have any COVID-19 symptoms:

- Fever or chills
- Difficulty breathing
- New or worsening cough
- Loss of smell, taste or appetite
- Sore throat
- Vomiting or diarrhea
- Aching throughout the body

Your safety and well-being are our top priority. If you have any of these symptoms, call 858-939-5400 to reschedule your surgery.

○

ARRANGE TRANSPORTATION:

For your safety, you must have someone drive you to and from surgery.

- Your companion will need to **drop you off** at the surgery center for safety reasons. You will be impaired from anesthesia and will not remember all instructions. You should anticipate staying a total of three hours. We will call your companion to pick you up from surgery.

- You will NOT be able to drive the day after surgery. Please plan to have someone drive you to your post-op eye exam.

- You will NOT be able to drive for at least seven days after surgery depending on how your eye heals.

1 DAY BEFORE SURGERY: ____________________

Use only your antibiotic (Ofloxacin) eye drops 4 times a day (every 4 hours while awake) in the eye that will have surgery. We will tell you how to use the other eye drops (Prednisolone) AFTER the surgery.

- If you feel sick or cannot come to surgery, please immediately notify our office at 858-939-5400

SURGERY DATE: ______________________

***Check-in Time*:** ______________________

Check-in Location: **Sharp Surgery Center**
3075 Health Center Drive
San Diego, CA 92123
→ **Admitting Desk:**

- **Do <u>NOT</u> eat** any solid food after 11:00PM the night before your surgery and until AFTER your surgery.
- You may drink only clear liquids (water, gatorade) up until 2 hours before your check-in time. Avoid carbonated beverages, dairy milk products, and alcohol.
- Please **TAKE** your blood pressure and heart medications with a tiny sip of water. Please withhold all other medications. If you have any questions, please check with your primary care doctor.
- Remove all eye makeup and lotion. Keep your face clean for surgery.
- Dress comfortably
- **<u>Place one drop</u>** of your antibiotic eye drop in your eye that will have surgery before you leave home.
- Bring your phone, photo ID, insurance card, and credit card for insurance co-pay, sunglasses and eye drops. Please leave jewelry & valuables at home.
- Bring **COMPLETED** White Health Questionnaire Form
- You will NOT be able to drive home after surgery. You will need someone to drive you home and pick you up 3 hours after your check-in time.

1 DAY AFTER SURGERY CHECK-UP: ________________

Check-in Time: ______________

Check-in Location: Sharp Surgery Center
3075 Health Center Drive
San Diego, CA 92123
Suite 401: 4th FLOOR

- Bring all your eye drop bottles & medication instructions sheets.

- Bring your current eyeglasses, sunglasses, insurance card, phone, and face mask with you.

- ***You will NOT be able to drive to this office appointment.*** Your eye will still be blurry and the anesthesia that you received yesterday has NOT cleared from your body. Please arrange for transportation to and from the office appointment for your safety and the general public.

- Please leave jewelry and valuables at home.
- Expect to be here for approximately 60 to 90 minutes for this appointment.
