# Supplementary material for: Digital Health Support for Cataract Surgery With the Sharp Health Companion CareKit App: Randomized Controlled Trial
Source: JMIR Mhealth Uhealth. 2026 Apr 29;14:e78710. doi: 10.2196/78710 (PMC13173071; doi:10.2196/78710)
Supplement: Multimedia Appendix 4 [file mhealth_v14i1e78710_app4.docx]

# Multimedia Appendix 3 - Patient Survey Questions

**Baseline Survey (Preoperative Office Consultation)**

1. Enter your study identifier number
2. What is your age?
3. What is your gender?

- Female
- Male
- Other
- Decline to state

1. What is your ethnicity? (Please select all that apply.)

- American Indian or Alaskan Native
- Asian or Pacific Islander
- Black or African American
- Hispanic or Latino
- White / Caucasian
- Prefer not to answer
- Other (please specify):

1. How does your iPhone PRIMARILY connect with the internet when you are at HOME?

- Home Wi-Fi
- Mobile cellular plan (AT&T, Verizon, T-Mobile, etc)
- I do not have internet access at home
- I do not know

1. How comfortable are you using apps on your smartphone? (Other than phone & text messaging)

- 1 - Least comfortable
- 2
- 3
- 4
- 5 - Most comfortable

1. Have you used any medical or health apps on a smartphone before? (For example: Follow My Health or Epic MyChart)

- Yes
- No

1. Do you use a smartwatch?

- Yes - Apple Watch
- Yes - Fitbit Versa or Fitbit
- Yes - Samsung Galaxy Watch
- No
- Other (please specify)

## Postoperative Day 1 Survey

1. Enter your study identifier number
2. Before your surgery, what did you use to help prepare for surgery?

- Used paper instructions
- Used Sharp Health Companion app
- Used BOTH Paper Instructions AND Sharp Health Companion App
- I did not review any information (paper instructions or Sharp Health Companion App)

1. When did you review your information (paper instructions and/or Sharp Health Companion App) before surgery?

- On the day of surgery
- 1 day before surgery
- 2 - 7 days before surgery
- 8 - 30 days before surgery
- >31 days before surgery
- I did NOT review any information before surgery

1. Before your surgery, did anyone in this surgeon's office give you all the information you needed about your eye surgery?

- Yes - definitely
- Yes - somewhat
- No

1. Before your surgery, did anyone in this surgeon's office give you easy to understand instructions about getting ready for your eye surgery?

- Yes - definitely
- Yes - somewhat
- No

1. Before your surgery, on a scale from 1 to 10, how would you rate your preparedness for surgery given the information you received from the surgeon’s office?

- 1 - Least prepared
- 2
- 3
- 4
- 5
- 6
- 7
- 8
- 9
- 10 - Most prepared

1. How would you rate your surgery experience so far with the information you’ve been provided?

- 1 - Least favorable
- 2
- 3
- 4
- 5
- 6
- 7
- 8
- 9
- 10 - Most favorable

1. If you used the Sharp Health Companion app, did you have to use the backup paper instructions at any time?

- Yes - definitely used the paper instructions
- Yes - somewhat used the paper instructions
- No - I did not have to use my backup paper instructions
- No - The Sharp Companion App was not available for me to use

1. How does the Sharp Health Companion App compare to other medical apps you have used?

- Easier & more valuable
- About as easy & valuable
- Not as easy or valuable

1. I would use the Sharp Health Companion App for other surgeries or medical procedures if available.

- Yes - definitely
- Yes - somewhat
- No

## Postoperative Month 1 Survey

1. Enter your study identifier number?
2. Before your surgery, did anyone in this surgeon's office give you all the information you needed about your eye surgery?

- Yes - definitely
- Yes - somewhat
- No

1. Before your surgery, did anyone in this surgeon's office give you easy to understand instructions about getting ready for your eye surgery?

- Yes - definitely
- Yes - somewhat
- No

1. Before your surgery, on a scale from 1 to 10, how would you rate your preparedness for surgery given the information you received from the surgeon’s office?

- 1 - Least prepared
- 2
- 3
- 4
- 5
- 6
- 7
- 8
- 9
- 10 - Most prepared

1. After your surgery, on a scale from 1 to 10, how would you rate your preparedness for your after surgery care instructions?

- 1 - Least prepared
- 2
- 3
- 4
- 5
- 6
- 7
- 8
- 9
- 10 - Most prepared

1. Did anyone in this surgeon’s office give you easy to understand instructions about what to do during your recovery period?

- Yes - definitely
- Yes - somewhat
- No

1. How would you rate your overall surgery experience from preparation to recovery using the information you were provided?

- 1 - Least favorable
- 2
- 3
- 4
- 5
- 6
- 7
- 8
- 9
- 10 - Most favorable

1. If you used the Sharp Health Companion App, did you have to use the backup paper instructions at any time?

- Yes - definitely used the paper instructions
- Yes - somewhat used the paper instructions
- No - I did not have to use my backup paper instructions
- No - The Sharp Companion App was not available for me to use

1. How does the Sharp Health Companion App compare to other medical apps you have used?

- Easier & more valuable
- About as easy & valuable
- Not as easy or valuable

1. I would use the Sharp Health Companion App for other surgeries or medical procedures if available.

- Yes - definitely
- Yes - somewhat
- No

1. How likely is it that you would recommend Sharp HealthCare to a friend or colleague based on your experience?

- 1 – Not at all likely
- 2
- 3
- 4
- 5
- 6
- 7
- 8
- 9
- 10 – Extremely likely
